# Microflora, Contents of Polyamines, Biogenic Amines, and TVB-N in Bovine Offal and Game Meat for the Raw-Feeding of Adult Dogs

**DOI:** 10.3390/ani13121987

**Published:** 2023-06-14

**Authors:** Sarah Lindinger, Susanne Bauer, Zuzana Dicakova, Brigitte Pilz, Peter Paulsen

**Affiliations:** 1 Unit of Food Hygiene and Technology, Institute of Food Safety, Food Technology and Veterinary Public Health, University of Veterinary Medicine Vienna, Veterinärplatz 1, 1210 Vienna, Austria; sarah.lindinger@vetmeduni.ac.at (S.L.); susanne.bauer@vetmeduni.ac.at (S.B.); brigitte.pilz@vetmeduni.ac.at (B.P.); 2 Department of Food Hygiene, Technology and Safety, University of Veterinary Medicine and Pharmacy in Košice, Komenskeho 73, 04181 Košice, Slovakia; zdicakova@gmail.com

**Keywords:** RMBD, red offal, pre-stomach, udder, microbiology, *Salmonella*, biogenic amines, total volatile basic nitrogen, spoilage

## Abstract

**Simple Summary:**

Raw-feeding of dogs has become popular, but there is limited awareness if such feedstuff is sold in a fresh or decomposed condition, and if bacteria pathogenic to dogs and humans are present. “Freshness” and numbers of bacteria were examined in 99 samples for the raw-feeding of adult dogs. Samples were displayed and bought in deep frozen (−20 °C) condition. The median number (with first and third quartiles in brackets) of bacteria was 7.4 [6.4; 8.0], 6.5 [5.5; 7.7], and 4.8 [3.9; 5.6] log CFU/g for the total aerobic bacteria of *Pseudomonas* and *Enterobacteriaceae*, respectively. In bovine “red offal” (*n* = 41), the numbers of bacteria were lower (*p* < 0,05) than in other offal (n = 46; pre-stomach, esophagus, udder, and mixes thereof) and wild game meat (*n* = 13). The concentration of amino acid breakdown products indicated some degree of decomposition; however, only 10.1% of samples were found to be not compliant with a maximum amine content proposed for pet food. However, merely 19.2% of samples complied to the EU microbiological requirements. The pathogenic bacterium *Salmonella* was recovered from 12.1% of samples. Whilst the risk of exposure of humans handling such pet food can be reduced by hygiene precautions, the risk remains that dogs can acquire a feed-borne salmonellosis and shed the pathogen as a result.

**Abstract:**

Microflora and contents of biogenic amines/polyamines and total volatile basic nitrogen (TVB-N) in 99 samples of bovine offal (red offal, *n* = 41 and other offal and mixes, *n* = 45) and wild game meat (*n* = 13) for raw meat-based diets (RMBD) for dogs were analyzed. Samples were bought in 11 local pet food shops and in one game-handling establishment in Austria (Lower Austria, Styria, and Vienna) in September and October 2022. Median contents (first and third quartiles in brackets) of cadaverine, histamine, tyramine, spermidine, and spermine were 20.7 [16.7; 28.6]; 25.4 [17.1; 47.2]; 18.9 [13.6; 38.9]; 15.2 [11.2; 21.2]; and 41.9 [<limit of detection; 64.5] mg/kg wet weight, respectively. The sum of putrescine + cadaverine + histamine + tyramine was >50 mg/kg in 85.9% of samples, indicating the use of low-quality ingredients or inappropriate storage conditions. However, only 10.1% of samples were determined to be not compliant with a maximum amine content proposed for pet food. Median contents of the total aerobic bacteria counts (TACs), *Pseudomonas*, and *Enterobacteriaceae* were 7.4 [6.4; 8.0]; 6.5 [5.5; 7.7]; and 4.8 [3.9; 5.6] log CFU/g, respectively, with significantly lower counts in red offal RMBD (*p* < 0.05). TVB-N exceeded 150 mg/kg in 87.9% of samples. The TACs and *Enterobacteriaceae* numbers in red offal RMBD were comparable to those in food-grade red offal after 6 days of aerobic storage at 7 °C, i.e., temperatures higher than required for food-grade offal, but acceptable for animal by-products intended for RMBD production. In 80.8% of samples, numbers of *Enterobacteriaceae* exceeded the EU legal limit. From 12 of these samples, Salmonellae was able to be isolated, with counts from 0.03 MPN/g to 110 MPN/g. *Salmonella enterica* ser. Montevideo (*n* = 3), and *S*. *enterica* ser. Give and *S. enterica ssp. Diarizonae* (*n* = 2 each) were the most frequently isolated, while *Listeria monocytogenes* was rarely recovered (2%). Whilst exposure of humans handling such pet food can be reduced by hygiene precautions, the risk remains that dogs can acquire a feed-borne salmonellosis and shed the pathogen.

## 1. Introduction

As many slaughter by-products do not enter the food chain for health, sensory, or commercial reasons, they are either disposed of or further processed, e.g., into pet food [1,2,3,4,5]. Beef processing generates a substantial amount of such products, amounting up to 44% of cattle live weight [6]. Pet owners can obtain these products either in processed or in raw form, with the latter for a raw meat-based diet (RMBD), often also termed “Biologically Appropriate Raw Food” (BARF). They give various reasons for why they decide to feed their pets in this way, be it the assumption that RMBD products are healthier, or that they are more species-appropriate compared to the conventional dry or wet pet food [7,8,9,10].

When processing animal by-products into pet food in the European Union, Regulation (EC) No 1069/2009 and Regulation (EC) No 142/2011 are applicable [11,12] in addition to the general food law [13]. In more detail, Regulation (EC) No 1069/2009 [11] defines three categories of animal by-products, with category 3 comprising “carcases and parts of animals slaughtered or, in the case of game, bodies or parts of animals killed, and which are fit for human consumption in accordance with Community legislation, but are not intended for human consumption for commercial reasons” and carcasses and other parts originating from animals that have passed the ante-mortem inspection and have been slaughtered in an approved slaughterhouse, but are either generally not fit for human consumption (e.g., pig bristles) or are “rejected as unfit for human consumption in accordance with Community legislation, but which did not show any signs of disease communicable to humans or animals” (Article 10, Regulation (EC) No 1069/2009 [11]. Such material can be used for the manufacturing of raw pet food under several precautions (Article 14, Regulation (EC) No 1069/2009); notably, raw feeding is not allowed for farm animals. Regulation (EC) No 142/2011 [12] lays down the hygiene requirements for such RMBD products, and in its Annex XIII, Chapter II, defines the microbiological criteria for the acceptance of feed batches. In addition, the pet food industry has established standards, and researchers have applied these standards for the foods of animal origin on the RMBD (details are presented in the context of this study in Section 2.3, Section 2.5, Section 4.1, and Section 4.3 of this article). Maximum temperatures for raw materials for the manufacture of a RMBD have been defined [12], and criteria for the “safe” feeding stuff must be fulfilled [13].

Although RMBD products are sometimes labelled or advertised as “complete feed”, they often do not provide a nutrient supply that fulfils all the requirements of growing or adult dogs [14,15]. In addition, it has been reported that RMBD products might not meet the hygienic quality standards set for pet food. While canned pet food is sterilized before being placed on the market, RMBD products are usually sold without having been preserved beforehand. Thus, RMBD products need to be stored under refrigeration or deep-frozen (−20 °C) and need to be fed to the dogs soon after thawing [3,4,5,16].

Numerous studies have investigated the microbiological characteristics of RMBDs, on the one hand to assess the hygienic conditions of the feedstuff [4,5,9,15,16,17,18,19,20,21], and on the other hand concerning the occurrence of (food-borne) biological hazards (in particular *Salmonella*, *Listeria monocytogenes*, *Campylobacter*, and pathogenic *E. coli*) [9,10,15,16,17,18,19,20,21,22,23,24,25,26,27,28,29,30,31,32] or parasites [3,18], and finally also in relation to the presence and possible transmission of antibiotic resistance [10,16,19,20,22,24,25,26,27,28,29,30,32,33]. In essence, these studies highlighted that a RMBD will likely contain pathogenic bacteria, and that these bacteria or commensals can carry antibiotic resistance genes. *Salmonella*, in particular, represents a health hazard not only for dogs consuming RMBDs, but also for pet owners feeding such raw diets [25]. Furthermore, the levels of spoilage or hygiene indicator for bacteria are not infrequently above the legal limits.

Chemical indicators, such as total volatile basic nitrogen (TVB-N) and biogenic amines (BA) can be used as complementary methods to assess the hygienic status by showing the degradation of the proteins or amino acids. Changes in the proteins and nitrogen-containing compounds caused by spoilage mechanisms result in the accumulation of volatile nitrogenous compounds, which are summarized under the term TVB-N [34]. As higher amounts of TVB-N result in changes in the flavor and color, TVB-N has therefore been commonly used as an indicator for the quality of seafood. Recently, there have also been efforts to use TVB-N as a standard quality parameter for meat. However, according to Bekhit et al. [34], using the commonly described threshold of ≤150 mg/kg without making a distinction for the different animal species may be insufficient, as there are species-specific initial conditions which could affect the change in the TVB-N values. Therefore, it would be appropriate to set specific limit values for each animal species [34,35].

Biogenic amines (BAs) are produced during the breakdown of amino acids and thus are well suited as spoilage indicators in proteinaceous foods [4,5,36,37]. The presence of significant amounts of biogenic amines not only affects the food quality, but also the health of consumers, including both humans and animals. In particular, the intolerance of dogs to alimentary histamine has been reported and studied [38,39,40]. The severity of these health issues depends on the amount of BAs ingested and individual susceptibility and can range up to circulatory disorders or even death [4,5,34]. The pet food industry has recognized histamine as a significant hazard [41].

Amongst the biogenic amines, polyamines (i.e., spermidine and spermine, and its precursor, putrescine) are polycationic substances, which normally stabilize DNA, are involved in apoptosis, and are abundant in metabolically active cells [42,43], but also in tumor cells [44]. Previous research has indicated an “anti-aging” effect of alimentary polyamines in mice [45].

Biogenic amine- and polyamine-based quality indices for assessing the decomposition of proteinaceous foods have been proposed [46,47], and for pet food a maximum amine content has been suggested [48].

The purpose of this study was to perform a comprehensive assessment of the hygiene quality profile of RMBD products from bovine offal and wild game muscle tissue purchased from east Austria. For the pathogen *Salmonella*, which is covered in microbiological criteria in the EU legislation on animal by-products [12], *Salmonella* levels were determined to provide an estimate of the dose of the pathogens ingested by dogs. Total aerobic bacteria counts and the numbers of *Enterobacteriaceae* were determined in aerobically stored red offal and results were compared with those in RMBD samples. This was done to estimate whether the bacterial numbers in RMBD could be sufficiently explained by a prolonged storage or inappropriate storage temperature of the raw materials before being processed into RMBDs.

## 2. Materials and Methods

### 2.1. Beef Offal and Wild Game Meat for Raw-Feeding

In September and October 2022, 99 samples were bought from eleven RMBD stores (representing 11 different RMBD producers) in Lower Austria, Styria, and Vienna and one game handling establishment in Lower Austria. The products chosen were intended for adult dogs and contained only offal from cattle (*n* = 86) or muscle tissue from hunted wild game (red deer and roe deer; *n* = 13). Amongst the 86 offal samples, 41 were composed of bovine red offal (heart, lung, liver, spleen, and kidney), 20 consisted of the bovine pre-stomach, and 10 samples were mixes of red offal and pre-stomach, respectively. Eight samples were composed of the pharynx, larynx, and esophagus and the remaining seven samples consisted of the udder. Details are given in Supplementary Table S1.

All samples were obtained in deep-frozen condition (−20 °C), vacuum-packaged, or filled in casings, with packaging sizes from 250–1000 g. Prior to examination, the samples were thawed in a refrigerator at +3 °C for 3–6 h.

### 2.2. Microbiological Examination

Total aerobic bacteria and numbers of *Enterobacteriaceae*, *Staphylococcus aureus,* coagulase-negative staphylococci, and *Pseudomonas* were determined using plating methods.

First, the entire sample was transferred to a sterile plastic bag, kneaded, and then a 25 g portion was taken under sterile conditions. This portion was homogenized at a 1:10 ratio in the maximum recovery diluent (OXOID CM733, Oxoid, Basingstoke, UK) for 60 s in a Stomacher-type lab blender (Interscience, St. Nom, France). A series of ten-fold dilutions was prepared in the maximum recovery diluent and a 0.1 mL aliquot was spread onto the surface of plate count (PC) agar plates (Merck 105463; Merck, Darmstadt, Germany) for total aerobic bacteria, with violet red bile glucose (VRBG) agar (Merck 110275; Merck, Darmstadt, Germany), for *Enterobacteriaceae* (incubation for 24 h at 37 °C), with Baird–Parker (BP) agar (Merck 105406 with supplement 103785) for *Staphylococcus aureus* (incubation for 48 h at 37 °C), and Glutamate-Starch-Penicillin agar (GSP; Merck 110230 with Penicillin G supplement; Sandoz, Kundl, Austria) for *Pseudomonas* (incubation for 72 h at 25 °C), respectively. The number of typical colonies was counted to calculate the number of colony-forming-units (CFUs) per gram of RMBD sample. Results were compared with the EU limits set out for raw materials for pet foods [12] and process hygiene criteria for foods [49]. Analyzes were performed in duplicate and the average of the two results was reported.

The presence of *Salmonella* was examined according to DIN EN ISO 6579-1 [50], with the reagents obtained from Merck. Suspected colonies on selective agars were sub-cultured and assessed for biochemical properties (Enteropluri system; Liofilchem, Roseto degli Abruzzi, Italy) as well as serologically (omnivalent serum; Sifin, Berlin, Germany). Confirmed Salmonellae were sent to the National Reference Centre for serotyping. In samples containing Salmonellae, their numbers were determined by a 3-tube most-probable-number (MPN) method. In brief, triplicates of 10, 1, 0.1, and—in sample L8—0.01 g of sample in buffered peptone water were all incubated for 24 hrs at 37 °C, whereafter the selective enrichment and plating on solid selective media was performed as described above [50]. From the numbers of *Salmonella*-positive results per sample size (i.e., 10 g, 1 g, 0.1, and 0.01 g, respectively), an index number was generated, and the most probable number was determined [51].

The presence of *Listeria* was examined by means of antigen-detection from cultural enrichment (VIDAS LDUO; Biomerieux, Marcy l’Etoile, France), with reagents and procedures performed as specified by the manufacturer. Testing for *Salmonella* and *Listeria* was conducted once per sample.

### 2.3. Determination of TVB-N

Total volatile basic nitrogen (TVB-N) was determined according to the Commission Implementing Regulation (EU) 2019/627 [52]. A 50 g portion of each (undiluted) sample was taken and pre-homogenized in a grinder (Grindomix 200; Retsch, Haan, Germany).

Then, 90 mL of 0.6 M perchloric acid (Roth, Karlsruhe, Germany) was added to a 10 g sample, and the sample was then homogenized with a high-speed blender for 2 min (Ultra-Turrax T25, Jahnke & Kunkel, Staufen i. B., Germany). The slurry was filtered through a folded paper filter (MN 615¼; Macherey-Nagel, Düren, Germany). Then, 50 mL of the homogenate was distilled in a Kjeldahl apparatus (Büchi, Flawil, Switzerland), with the addition of 6.5 mL of sodium hydroxide solution and 0.05 mL anti-foaming agent into 100 mL of boric acid solution (Roth). The absorbed bases were titrated with 0.01 M hydrochloric acid to determine the content of volatile nitrogen. In addition, a blank test was performed using perchloric acid solution instead of the sample extract. Total volatile basic nitrogen was calculated according to the Commission Implementing Regulation (EU) 2019/627 [52] as mg N/100 g (Equation (1)).

(1) $$TVB-N=\frac{\left(V_{1}-V_{0}\right)*0.14*2*100}{M}$$

where:

*V*_1_ = volume of 0.01 M hydrochloric acid in ml for sample;

*V*_0_ = volume of 0.01 M hydrochloric acid in ml for blank;

*M* = mass of sample in g.

Analyzes were performed in duplicate and the average of the two results was reported in mg/kg wet weight.

### 2.4. Determination of Biogenic Amines and Polyamines (Cadaverine, Histamine, Putrescine, Tyramine, Spermidine, and Spermine)

From the pre-homogenized sample (Section 2.3), a 10 g portion was taken and mixed with 90 g 10% trichloroacetic acid. The suspension was homogenized for 60 s (Ultra-Turrax T25). The slurry was first filtered through a folded paper filter, and then through a 0.45 µm cellulose-acetate membrane filter (Roth). The filtrate was adjusted to a pH of 11 ± 0.1 with the addition of NaHCO_3_ solution and then reacted with dansyl chloride in the dark at 70 °C for 10 min (water bath). The liquid was taken to dryness under a reduced pressure (Rotavapor; Büchi) and the dried residue was washed with 2 mL acetonitrile to dissolve the dansyl derivatives of the amines [4,5]. These were subsequently separated on a RP-C_18_-HPLC column (Symmetry, 4.6 × 150 mm; 3.5 µm; Waters, Milford, MA, USA) with a gradient elution program [4,5]. Amine derivatives were detected by UV-absorption at 254 nm, and then identified and quantified by the external standard method. Analyzes were conducted in duplicate and the average of the two results was reported.

The HPLC apparatus and detector were from Waters company (models 2695 and 996, respectively). Amine standard substances and dansyl chloride were obtained from Sigma–Aldrich (St. Louis, MO, USA), while the other reagents and eluents/solvents were purchased from Thermo Fisher (Waltham, MA, USA). The limit of detection was 1.5–2.8 mg/kg, depending on the amine [4,5]. Results were expressed as mg/kg wet weight.

### 2.5. Biogenic Amine Index Values and Proposed Limits

The biogenic amine index (BAI) and an index value which focuses on mono- and diamines were calculated according to [46] (Equation (2)) and [53] (Equation (3)), respectively, to evaluate the amino acid decomposition of the samples.

(2) $$\mathrm{BAI}\;=\frac{\mathrm{putrescine}+\mathrm{cadaverine}+\mathrm{histamine}}{\mathrm{spermidine}+\mathrm{spermine}+1}$$

Amines in mg/kg wet weight; a BAI value > 1 is indicative for deterioration/decomposition [46].

(3) Index =putrescine+cadaverine+histamine+tyramine

Values in mg/kg wet weight; an index value exceeding 50 mg/kg is indicative for deterioration [53].

For pet food, a maximum level of 300 mg/kg for the sum of all amines was proposed [48] and histamine levels should not exceed 500 mg/kg and above [41].

### 2.6. Samples for the Storage Trial

Bovine edible offal (tongue, esophagus, heart, lung, diaphragm, liver, spleen, and kidney) was provided by a slaughterhouse. There, offal had been cooled to an internal temperature <3 °C (required maximum temperature for offal [54]) overnight. Offal samples were then transported under refrigeration to the laboratory and stored at 3 °C (maximum temperature for edible offal; simulating good practice) and 7 °C (maximum temperature for red meat muscles [54] and for animal by-products intended for RMBD production [12]) for 3 and 6 days under aerobic conditions, respectively in order to study the microbiota. For each type of offal, 15 samples (i.e., parts of ca. 250 g weight taken from 3–4 organs) were randomly assigned to 5 groups / sampling points (after cooling, i.e., 24 hrs after slaughter; after 3 and 6 days of storage at 3 and 7 °C, respectively). Samples were placed in plastic bags which remained open to allow the aerobic conditions to mimic storage conditions in practice. At each sampling point, the three samples were assessed for total aerobic bacteria and the numbers of *Enterobacteriaceae*. We report the averages of the log-transformed results for all offal samples (8 × 3 = 24) for each time–temperature combination.

### 2.7. Statistical Procedures

For the 99 RMBD samples, the results were compared to reference values derived from the legislation on animal by-products [12], foods [49,55,56], or recommendations from science [45], or the industry [41,48]. For comparing the numbers of bacteria, TVB-N, and amine contents and index values, bovine offal samples and wild game muscle tissue samples were considered separately. Within the offal group, we separated the red offal samples from the other offal. For calculation of the BAI, results below the limit of detection were set to “1”. The three product groups were also tested for statistically significant differences in regards to their compliance with the legal limits (3-by-2 chi-square test; MS Excel, with critical values obtained from [57]). Differences between the product categories in terms of the numbers of bacteria, TVB-N, and amine contents and index values were evaluated using non-parametric tests (Kruskal–Wallis test; MS Excel [57]), with *p* < 0.05 indicating a statistically significant difference.

For food-grade bovine offal samples, the influence of storage time and temperature on total aerobic bacteria or *Enterobacteriaceae* were assessed by the two-factor ANOVA with the Scheffé´s post-hoc test (Statgraphics 3.0 software). Results obtained after 6 days storage at 7 °C were compared to the results obtained for the RMBD samples.

## 3. Results

### 3.1. RMBD Samples

#### 3.1.1. General Microbiological Condition of RMBD Samples

The median values were 7.4; 6.5; 4.8; and 4.3 log CFU/g for the TAC, *Pseudomonas*, *Enterobacteriaceae*, and coagulase-negative staphylococci, respectively. For these groups of bacteria, “red offal” displayed significantly lower numbers than “other offal” and “wild game meat”. *Staphylococcus aureus* was detected in 45/99 samples, with a maximum value of 4.8 log CFU/g. The frequencies of samples with *Staphylococcus aureus* counts above the limit of detection (i.e., 2 log CFU/g) in “red offal”, “other offal”, and “wild game meat” were found to not differ significantly (*p* > 0.05).

The TAC exceeded 7 log CFU/g in 62/99, and 8 log CFU/g in 27/99 samples, respectively. Detailed results are displayed in Table 1.

*Salmonella* sp. was recovered in 12/99 samples (25 g aliquots). The isolates were serotyped by the National Reference Centre: *S. enterica* ser. Montevideo (three isolates), *Salmonella enterica* group IIIb and S. *enterica* ser. Give (two isolates each); and one isolate each was typed as *S*. *enterica* ser. Typhimurium, *S*. *enterica* ser. Infantis, *S*. *enterica* ser. Bovismorbificans, *S*. *enterica* ser. Dublin, and *S*. *enterica* ser. Stockholm, respectively. The determination of the MPN of *Salmonella* in these 12 samples yielded results in the range from <0.03/g to 110/g (Table 2). The frequencies of *Salmonella*-positive samples in “red offal”, “other offal” and “wild game meat” were found to not differ significantly (*p* > 0.05).

*Listeria monocytogenes* antigens were detected in two samples (larynx-esophagus and pre-stomach), whereas antigens from Listeriae other than *L. monocytogenes* were detected in 24 samples (twelve in red offal, nine in other offal, and three in wild game meat, respectively). The frequencies of *Listeria*-positive samples in “red offal”, “other offal”, and “wild game meat” were found to not differ significantly (*p* > 0.05).

#### 3.1.2. Contents of TVB-N, Biogenic Amines, and Polyamines of the RMBD Samples

Contents of TVB-N and amines are presented in Table 3 and Table 4. Statistically significant differences were observed only for the contents of cadaverine (which was found to be higher in the wild game muscle tissue) and for spermine (which was found to be lower in the wild game muscle tissue).

#### 3.1.3. Compliance of the Results with the Legal Requirements for RMBD Feed

Commission Regulation (EU) No 142/2001 [12] requires that in none of the five samples randomly taken during production or storage (before dispatch) can Salmonellae be detected in 25 g aliquots, and that numbers of *Enterobacteriaceae* are always less than 5000 CFU/g. Otherwise, the batch was considered as “unsatisfactory”. Only one sample was tested per batch, and not before dispatch, but at retail. However, since the feed items were displayed and sold deep-frozen, it can be assumed that the microbiological condition at the time of analysis reflected the condition before dispatch. Out of the 99 samples, only 19 fulfilled the criteria (see Table 5).

#### 3.1.4. Compliance of the Results with the Recommended Limits for Feed and Foods

In 89/99 samples, the sum of all amines was below a recommended limit of 300 mg/kg [48]. Histamine contents were always <500 mg/kg [41].

When applying the EU process hygiene criteria for fresh (=raw) minced meat [49], 33/99 samples complied with the upper limit “M” for total aerobic bacteria, and the TACs of 37/99 samples were <7 log CFU/g, a value commonly associated with the spoilage of fresh meat [58]. Frequency of samples with a TAC <6.7 and <7.0 log CFU/g was significantly higher in “red offal” than in the two other product groups (Table 6).

The number of samples complying with the limits for biochemical indicators was found to be in the same range, albeit with no statistical significance observed between the product groups. Although these limits do not directly apply to animal by-products for raw feeding, they do give an indication on the hygienic condition of the materials used.

### 3.2. Bovine Slaughter By-Products and Microbiological Condition after 3 and 6 Days of Aerobic Storage

The total aerobic bacteria counts of bovine offal were in the range of 4 log CFU/g at the day after slaughter, which increased to 4.8 log CFU/g when stored aerobically 6 days at 3 °C. When samples were stored at 7 °C, these numbers increased to 6.7 log CFU/g. Both the time and temperature, as well as the interaction of temperature with time were found to be statistically significant factors for the increase of the TAC. In contrast, numbers of *Enterobacteriaceae* were found to be influenced by the temperature and the interaction of temperature with time (Table 7). The averages reported for 6 days storage at 7 °C match well with the median values we found for “red offal” RMBD feed.

## 4. Discussion

### 4.1. Legislative Requirements

Reg (EC) Nr. 142/2011 [12] sets out the limits for feedstuff from animal by-products intended for the raw-feeding of pets. Although these limits apply to feedstuff before being placed on the market, it is also reasonable to use these limits for frozen RMBDs in retail. *Salmonella* was recovered from 12% of the samples. This was higher than the results for muscle-based RMBDs [21] in Austria (7.3%). Studies from different European countries reported a prevalence range from 2 to 71% [15,18,19,23,30,31]. The higher percentage of *Salmonella*-positive (offal) samples in our study compared to that of Koch et al. [21] (which assessed muscle-based samples obtained from bovines) raises the question of whether the use of (green) offal compared to muscle tissue is a significant risk factor for the presence of *Salmonella* sp.; also see Section 4.2. Further studies may also focus on this issue.

In 81% of the samples, *Enterobacteriaceae* levels exceeded the “M” limit of 5000 CFU/g [12]. Similar figures have been reported in studies from Austria [21] and Italy [15]. Likewise, in studies investigating coliforms instead of *Enterobacteriaceae*, numbers < 5000 CFU/g were the exception rather than the rule [19,23,31]. It has been reported that RMBDs from rabbit and poultry can contain higher numbers of *Enterobacteriaceae* [15], with maximum values exceeding 7 log CFU/g. Although, in our study, while the maximum was 7.6 log CFU/g, only 1/99 samples exceeded 7 log CFU/g, compared to 5/37 reported in a study from Italy [15].

### 4.2. Salmonella Serovars and Their Contents in the Samples

The *Salmonella* that had been recovered from twelve RMBD samples belonged to the species *enterica*. Furthermore, besides *S. enterica ssp. diarizonae*, seven different *S. enterica ssp. enterica* serovars were also identified.

*S*. *enterica* ser. Montevideo was isolated from three samples, with two containing pre-stomach tissue. Although the samples originated from two different retailers (corresponding to two separate RMBD producers), it cannot be excluded that there was a common source (i.e., that the two retailers obtained the raw material from the same slaughterhouse). Likewise, our results do not distinguish *Salmonella* presence due to infection/colonization of the live animals from contamination events during or post-evisceration. *S*. *enterica* ser. Montevideo has been reported in beef carcasses and ground beef [59], and also in RMBDs [17,28]. In the EU Zoonoses Report for 2021 [60], this serovar accounted for <1% of the reported *Salmonella* findings in bovines; however, there is no EU-wide harmonized testing scheme and no baseline study on the actual prevalence of *Salmonella* and its serovars in bovines in the EU [60].

In two samples, *S*. *enterica ssp. diarizonae* was identified. While to our knowledge there are no reports regarding the presence of this *Salmonella* subspecies in RMBDs, cases of infected sheep flocks and consequently contaminated sheep meat have been reported from various countries across Europe [61,62,63]. *S*. *enterica* ser. Give was also identified in two samples. This serovar has, albeit rarely, been isolated from bovines in the EU [60].

*S*. *enterica* serovars Typhimurium, Infantis, Bovismorbificans, Dublin, and Stockholm were found in one sample each. *S*. *enterica* ser. Typhimurium and *S*. *enterica* ser. Infantis are detected in RMBDs regularly [21,22,26,32]. Reports of the detection of *S*. *enterica* ser. Bovismorbificans in bovines, bovine meat, or RMBDs have been rare, but in 2021, this serovar was one of the 20 most frequently found serovars in humans infected with *Salmonella* [60].

In 2021, *S*. *enterica* ser. Dublin represented the most frequently found serovar in bovines in the EU (31%), followed by *S*. *enterica* ser. Typhimurium (30%). Being a cattle-adapted serovar [64,65], infections in humans with *S*. *enterica* ser. Dublin are infrequent but can cause severe disease, including septicemia and even death [65,66,67].

*S*. *enterica* ser. Infantis in turn was responsible for around 1.5% and 2% of the reported *Salmonella* infections in bovines and humans, respectively [60].

Reports of *S*. *enterica* ser. Stockholm infections seem to be rare. Gobeli-Brawand et al. reported that the serovar was detected in Switzerland for the first time in 2015 [68]. Since then, only two reports on *S*. *enterica* ser. Stockholm infections identified in an Indian slaughterhouse and in a human stool sample were mentioned in that study [69,70]. However, this finding underpins the zoonotic potential risk of this serovar.

Regarding RMBD, several authors have mentioned *S*. *enterica* ser. Heidelberg, *S*. *enterica* ser. Kentucky, and *S*. *enterica* ser. Saintpaul to be common serovars being shed from dogs fed with a RMBD [27,71]. However, these serovars were not found in our study.

In some studies, the prevalence of *Salmonella* on the surface of carcasses was investigated [72,73], but only limited data could be found on the prevalence of *Salmonella* in beef offal in particular [74,75,76]. McEvoy et al. investigated the prevalence of *Salmonella* in bovine carcasses, rumen, and feces and identified *S*. *enterica* ser. Dublin, *S*. *enterica* ser. Typhimurium, and *S*. *enterica* ser. Agona, respectively [74]. These results match with the findings in our study, as we also found *S*. *enterica* ser. Typhimurium and *S*. *enterica* ser. Dublin in samples of rumen and omasum, respectively. McEvoy et al. suggested that fasting prior to slaughter may cause a higher prevalence of *Salmonella* in the pre-stomach because of the fasting inducing lower concentrations of volatile fatty acids, which normally inhibit *Salmonella* growth [74]. In contrast, Im et al. suspected that the improper handling of (green) offal could lead to a higher prevalence of *Salmonella* in green offal compared to meat products intended for human consumption [76].

In the studies mentioned above, only the presence and absence of *Salmonella* was examined. From examining the available literature, we found a few additional studies investigating levels of *Salmonella* in poultry and pork [77,78] and rendered products [79], but not specifically in RMBD products. The fact that six of our samples contained >1 *Salmonella*/g indicates that for these samples, ingestion of a 100–200 g portion results in the intake of several hundred to several tens of thousands of the pathogen per meal.

### 4.3. General Microbiological Condition and Chemical Freshness Indicators

The numbers of total aerobic bacteria in our samples were in the upper expected range and were comparable to those reported by Koch et al. [21], Solis et al. [9], and Vecciato et al. [15], respectively. Somewhat lower numbers with the maxima well below 7 log CFU/g were reported by Van Bree et al. [18] and Bottari et al. [31], respectively. Although the number of total aerobic bacteria in RMBD is per se not a convincing indicator of spoilage [15], the number of *Pseudomonas* (median 6.5 log CFU/g) in our samples indicates some degree of proteolytic activity. Coagulase-negative staphylococci were recovered from 45/99 samples, with a maximum of 4.8 log CFU/g. Nine samples exceeded 5000 CFU/g, which is the alert level for fresh minced meat suggested by German food safety experts [80]. *Listeria monocytogenes* was recovered in 2/99 samples, which is low compared to other studies [16,18,21,31].

In regard to the contents of biogenic amines, the median contents of cadaverine, histamine, tyramine, and spermidine in wild game meat (36; 19.5; 29.8; and 14.3 mg/kg wet weight, respectively) roughly complied with those previously reported for RMBDs from bovine muscle tissue (8.3; 18.6; 16.1; 21.1; 17.4) [21]. A significantly higher content of spermine and a lower content of its precursor putrescine could be explained by the different metabolic activities of the inner organs resulting in the generally higher concentrations of the polyamines in the inner organs than in the muscle tissues [81]. Contents of amines other than polyamines are largely attributable to the metabolic activity of contaminant bacteria, and may therefore, differ according to microbial contamination and storage conditions [36,37]. The accumulation of biogenic amines usually occurs when bacterial numbers exceed 7 log CFU/g [82]. Since this was the case for the majority of the RMBD samples, it was not surprising that the calculation of the index values showed that protein/ amino-acid degradation had occurred in a large fraction of samples. Likewise, median TVB-N values in most samples were above the acceptable limits for muscle tissue [55,56]. Admittedly, such limits must be applied with caution when the initial (“natural”) contents of the food or feed item are not known [35].

### 4.4. Comparison of RMBD Samples with Bovine Offal Stored at Elevated Temperature (7 °C) Conditions

RMBDs can be produced from food-grade meat or from food-grade slaughter by-products or animal by-products (category 3 material according to the EU Regulation 1069/2009 [11]. For food-grade bovine offal, bacteria numbers after 3 days of storage at 3 °C (4.7 log CFU/g for total aerobic bacteria and 2.3 log CFU/g for *Enterobacteriaceae*), i.e., under conditions typical in food retail, are much lower than for the tested RMBD samples.

Depending on the nature of the organ/tissue, the contamination events, and the conditions of storage, microbiological conditions can significantly vary, e.g., low numbers of bacteria in RMBDs have been reported by van Bree et al. [18] and Bottari et al. [31]; and the influence of partial defrosting during the delivery of deep-frozen RMBDs has been investigated by Vecchiato et al. [15]. However, there is no clear picture as to whether the raw materials at the slaughterhouse or their “history” in terms of their storage conditions account for the high or low numbers of bacteria found in RMBDs in retail. The usefulness of longitudinal monitoring along the RMBD chain has been mentioned [21], but studies on this are lacking. In the experiment with food-grade bovine offal, it was shown that even a moderately elevated storage temperature (7 °C) for 6 days aerobic storage yielded total aerobic bacteria and *Enterobacteriaceae* counts as found in the red offal RMBD samples (for which 7 °C would be an appropriate storage temperature [12]). In other words, processing of food-grade offal with an undue delay could result in RMBD products with much lower numbers of TAC.

Admittedly, this does not resolve the *Salmonella* feed safety issue.

### 4.5. Implications for Dogs´ and Humans´ Health

According to the Zoonoses Report of the European Union, *S. enterica* ser. Enteritidis, *S. enterica* ser. Typhimurium, and monophasic *S. enterica* ser. Typhimurium were the predominant serovars in reported cases of human salmonellosis in the European Union in 2021 [60].

A few of the additional serovars found in our study (*S*. *enterica* ser. Infantis, *S*. *enterica* ser. Montevideo, *S*. *enterica* ser. Bovismorbificans, and *S*. *enterica* ser. Dublin, respectively) also occur in the list of the 20 most frequent serovars causing human salmonellosis in the European Union in the year 2021 [60].

Looking at the results of various studies from 2015–2021, the *Salmonella* prevalence in dogs ranges from 0.23 to 12.86%, with *S*. *enterica* ser. Newport, *S*. *enterica* ser. Enteritidis, and *S*. *enterica* ser. Typhimurium being the most predominantly identified serovars [83].

As has been known for many years, dogs rarely become clinically ill, but are much more likely to be asymptomatic carriers of *Salmonella* [84,85], and can shed the pathogen for up to six weeks [24]. In 2022, Russini et al. published a study on *S*. *enterica* ser. Typhimurium infections in children, in which the family dogs were identified as asymptomatic carriers [86], but it was not specified in that study whether the dogs were fed a RMBD or a conventional diet.

However, cases of clinical salmonellosis in dogs have also been reported and include symptoms such as a fever, diarrhea, and abdominal cramps [24,87].

A study from 2017, where dogs were tested for *Salmonella* showed that there were more *Salmonella*-positive dogs fed a RMBD than *Salmonella*-negative dogs fed a RMBD (16.7% and 7.2%, respectively), which further confirms the risk of a RMBD with respect to *Salmonella* [87].

In addition to the pets themselves in their role as carriers, the improper handling of contaminated (raw) pet food poses a major risk to the pet owner, especially when the pet food is prepared in the owner´s kitchen [16,87,88]. This is aggravated by the fact that, in at least some countries, the proportion of *Salmonella*-positive samples in feeding stuff and, in particular, in raw-meat-based pet food, is increasing [89]. Still, source attribution in human cases is far from complete [60], which means that the role of RMBDs in human infections, or a possible trend towards increased human cases due to the handling of RMBDs cannot be assessed properly.

The amount of *Salmonella* that must be ingested to cause infection depends on several factors, including serovar, food source and handling, and the general state of the health of the individual [83]. Only limited data on infectious doses of the individual *Salmonella* serovars was available. In a study by Hara-Kudo and Takatori, *S*. *enterica* ser. Montevideo and *S*. *enterica* ser. Agona were identified as the serovars with the lowest ingestion doses leading to an infection with the levels of 363 MPN and <1500 CFU, respectively, compared to, for example, *Salmonella enterica* ser. Enteritidis with doses of at least 3.51 × 10^6^ CFU [83,90]. However, the infection rates in these outbreaks were relatively low at 12.5% and 23.6% for *S*. *enterica* ser. Montevideo and *S*. *enterica* ser. Agona, respectively, indicating that infection does not only depend on the serovar and number of Salmonellae ingested [83,89]. Regarding *S*. *enterica* ser. Montevideo, ingestion of a 150 g portion of sample H14 results in the uptake of ca. 450 cells, which is in the infectious range for dogs [90].

## 5. Conclusions

The analysis of 99 pet food samples for raw feeding of dogs revealed a poor compliance with the EU legal limits for such feedstuff. Since the samples were sold deep-frozen and in sealed casings, it can be assumed that this non-compliance already had existed at the production site. The presence of Salmonellae in quantities of >1 MPN/g in six samples, with a maximum of 110 MPN/g raises concerns about a food-borne transmission and the subsequent intestinal colonization of dogs. Likewise, the storage and handling of such food, as well as the cleaning of dishes requires hygiene precautions to protect humans [91]. Altogether, it is questionable whether the raw-feeding-related health risks to dog owners and dogs can be managed sufficiently.

The numbers of total aerobic bacteria and *Pseudomonas* indicate that these samples had a limited shelf life after defrosting. The high numbers of bacteria may be typical of the organ (e.g., green offal), or be a result of contamination or inappropriate storage. Even food-grade red offal can reach high bacterial numbers when stored 6 days at a moderately elevated temperature of 7 °C. Indicators for amino acid decomposition provide additional information on the “freshness” of raw meat-based pet food. A thorough analysis of the cold chain from slaughter to the manufacture of RMBDs will be the subject of future studies.

## Figures and Tables

**Table 1:** Total aerobic bacteria, *Enterobacteriaceae*, *Pseudomonas*, coagulase-negative staphylococci and *Staphylococcus aureus* in raw meat-based diet samples.

	Total Aerobic Bacteria, Log CFU/g	*Enterobacteriaceae*, Log CFU/g	*Pseudomonas*, Log CFU/g	Coagulase-Negative Staphylococci, Log CFU/g	*Staphylococcus aureus* *
Red offal	6.5 ^a^ [6.1; 7.4]	4.1 ^c^ [3.3; 4.9]	5.8 ^e^ [5.3; 6.9]	3.7 ^g^ [3.3; 4.5]	20/41; max: 4.8 log CFU/g
Other offal and mixes	7.7 ^b^ [7.0; 8.1]	5.1 ^d^ [4.0; 5.7]	7.2 ^f^ [5.8; 7.9]	4.7 ^h^ [4.2; 5.6]	21/45; max: 4.7 log CFU/g
Game meat (muscle)	7.6 ^b^ [7.4; 7.9]	5.5 ^d^ [5.3; 5.8]	7.3 ^f^ [7.1; 7.8]	4.1 ^h^ [3.8; 4.4]	4/13; max: 4.0 log CFU/g
All samples	7.4 [6.4; 8.0]	4.8 [3.9; 5.6]	6.5 [5.5; 7.7]	4.3 [3.6; 5.1]	45/99; max: 4.8 log CFU/g

Data refer to the wet weight and are presented as medians with the 1st and 3rd quartiles in brackets; * for *Staph. aureus*, the frequency of samples with results above the limit of detection (i.e., >2 log CFU/g), and the maximum value are reported. Within the columns, different superscripts indicate statistically significant differences, *p* < 0.05.

**Table 2:** The number of Salmonellae, in MPN/g, in raw meat-based diet samples that had tested positive for *Salmonella* sp. In 25 g aliquots.

Sample Number	Type	*Salmonella enterica* Serovar (ser.) or Subspecies (*ssp.*)	MPN/g
B1	spleen	*ssp. Diarizonae* (IIIb)	0.03
B3	pre-stomach (omasum)	ser. Montevideo	0.15
D7	mix * (various offal)	ser. Infantis	0.03
D9	pre-stomach (rumen, omasum)	*ssp. Diarizonae* (IIIb)	0.03
F1	pre-stomach (omasum)	ser. Dublin	4.6
F4	mix (rumen and esophagus)	ser. Stockholm	2.1
H1	pre-stomach (rumen)	ser. Bovismorbificans	2.1
H6	lung	ser. Montevideo	0.15
H14	wild game meat	ser. Montevideo	2.9
L8 *	pre-stomach (rumen)	ser. Typhimurium	>11/110 *
O1	mix (esophagus and other offal)	ser. Give	0.092
O9	lung	ser. Give	4.6

Letters in the first column indicate the manufacturer; * First result refers to a 3-tube MPN with 10/1.0/0.1 g, whereas the second result refers to a 3-tube MPN with 1.0/0.1/0.01 g.

**Table 3:** Contents of TVB-N and biogenic amines in raw meat-based diet samples.

	TVB-N, mg/kg	Cadaverine, mg/kg	Histamine, mg/kg	Tyramine, mg/kg
Red offal	262 [227; 348]	17.5 ^a^ [<LOD; 23.5]	23.5 [18.0; 85.0]	17.5 [11.1; 36.3]
Other offal and mixes	253 [180; 393]	20.8 ^a^ [17.7; 30.6]	29.1 [17.3; 45.5]	20.2 [13.9; 30.8]
Game meat (muscle)	313 [272; 353]	36.0 ^b^ [21.3; 53.8]	19.5 [15.0; 29.7]	29.8 [18.7; 63.4]
All samples	27.2 [19.4; 36.9]	20.7 [16.7; 28.6]	25.4 [17.1; 47.2]	18.9 [13.6; 38.9]

Data refer to the wet weight and are presented as medians with the 1st and 3rd quartiles in brackets. Within the columns, different superscripts indicate statistically significant differences, *p* < 0.05; LOD = limit of detection.

**Table 4:** Contents of polyamines in raw meat-based diet samples and index values.

	Putrescine, mg/kg	Spermidine, mg/kg	Spermine, mg/kg	BAI	Sum of Four Amines *, mg/kg	Sum of All (Six) Amines, mg/kg
Red offal	<LOD [<LOD; 20.6]	18.5 [12.1; 22.8]	57.4 ^a^ [<LOD; 82.2]	1.0 [0.6; 1.9]	90.4 [50.9; 173.1]	135.5 [84.7; 227.2]
Other offal and mixes	<LOD [<LOD; 26.9]	14.8 [11.2; 21.2]	37.8 ^a^ [<LOD; 61.9]	1.3 [0.8; 2.3]	88.2 [70.7; 120.2]	133.9 [100.8; 187.7]
Game meat (muscle)	16.9 [<LOD; 24.3]	14.3 [10.6; 15.5]	<LOD ^b^ [<LOD; 41.9]	1.9 [1.1; 4.2]	123.3 [75.4; 155.8]	128.3 [111.9; 166.6]
All samples	<LOD [<LOD; 24.9]	15.2 [11.2; 21.2]	41.9 [<LOD; 64.5]	1.3 [0.8; 2.3]	95.9 [67.8; 155.8]	134.9 [98.1; 206.6]

Data refer to the wet weight and are presented as medians with the 1st and 3rd quartiles in brackets; * putrescine + cadaverine + histamine + tyramine. Within the columns, different superscripts indicate statistically significant differences, *p* < 0.05; LOD = limit of detection.

**Table 5:** Compliance of the 99 raw meat-based diet samples, according to the product type and supplier.

Supplier	Pre-Stomach	Larynx and Esophagus	Udder	Red Offal	Mixes	Game Meat (Muscle)	Non-Compliant/Tested
B	1/1 *			**3/3**	1/1		5/5
C	1/1	1/1			1/1		3/3
D	**1/1**			2/6	**2/2**		5/9
E	0/2	3/3		3/3			6/8
F	**2/2**	1/1			**1/1**	2/2	6/6
H	**4/4**			**6/6**		**4/4**	14/14
K						1/1	1/1
L	**¾**	1/1	2/2	5/7		1/1	12/15
N	1/1	2/2	3/3	1/1	1/1	2/2	10/10
O	1/1		1/1	**3/6**	**2/2**	2/2	9/12
P	2/2			1/6	1/1		4/9
S	1/1		1/1	3/3	0/1	1/1	6/7
total	15/20	8/8	7/7	27/41	9/10	13/13	80/99

Letters in the first column indicate the manufacturer; in the second to last column, the number of samples non-compliant to the *Enterobacteriaceae* criterion (i.e., ≥ 5000 CFU/g) is given, followed by the total of samples tested. * Bold lettering indicates that one of the samples contained *Salmonella* in a 25 g aliquot.

**Table 6:** Compliance of TVB-N and amine contents in raw meat-based diet samples with the recommended limits for feed and food.

	TVB-N < 150 mg/kg	TVB-N < 200 mg/kg	BAI < 1	Sum of Amines ** < 50 mg/kg	Sum of all Amines < 300 mg/kg	TAC < 6.7 log CFU/g	TAC < 7 log CFU/g
Red offal	2/41 *	10/41	20/41	3/41	43/45	23/41 ^a^	24/41 ^d^
Other offal and mixes	8/45	15/45	15/45	10/45	33/41	10/45 ^b^	12/45 ^e^
Game meat (muscle)	2/13	3/13	2/13	1/13	13/13	0/13 ^c^	1/13 ^f^
All samples	12/99	28/99	37/99	14/99	89/99	33/99	37/99

Results refer to the wet weight; * Number of compliant samples/total number of samples; ** putrescine + cadaverine + histamine + tyramine. Within the columns, different superscripts indicate statistically significant differences, *p* < 0.05.

**Table 7:** Total aerobic bacteria counts (TACs) and *Enterobacteriaceae* in bovine offal, after aerobic cold storage (1, 3, and 6 days, respectively), in log CFU/g.

	1d-3 °C	3d-3 °C	6d-3 °C	3d-7 °C	6d-7 °C
TAC	4.1 ± 0.5 ^Aa^	4.7 ± 1.0 ^Bb^	4.8 ± 0.8 ^Bb^	5.3 ± 0.8 ^Cc^	6.7 ± 1.1 ^Cc^
*Enterobacteriaceae*	2.3 ± 0.6 ^Dd^	2.3 ± 0.5 ^De^	2.7 ± 0.7 ^De^	3.1 ± 0.9 ^Df^	4.0 ± 1.3 ^Df^

Results refer to the wet weight; Data are averages ± standard deviation of 24 samples (8 types of offal in triplicate). Within the rows, different capital superscripts indicate statistically significant differences by time, whereas lowercase superscripts indicate statistically significant differences by temperature.

## Data Availability

Original data are available at: https://phaidra.vetmeduni.ac.at/o:1582 (accessed on 11 June 2023).

## Supplementary Materials

The following supporting information can be downloaded at: https://www.mdpi.com/article/10.3390/ani13121987/s1, Table S1: Number of samples according to organ/tissue and supplier.
