# Supplementary material for: Microflora, Contents of Polyamines, Biogenic Amines, and TVB-N in Bovine Offal and Game Meat for the Raw-Feeding of Adult Dogs
Source: Animals (Basel). 2023 Jun 14;13(12):1987. doi: 10.3390/ani13121987 (PMC10295124; doi:10.3390/ani13121987)
Supplement: Supplementary file 1 [file animals-13-01987-s001.zip › animals-2398007-supplementary.pdf]

animals

MDPI

SUPPLEMENTARY FILE

Table S1. Number of samples according to organ/tissue and supplier.

supplier*	tongue	lung	heart	liver	spleen	kidney	sum of (red offal)	layrynx, esophagus	rumen	omasum	udder	mix	sum of (other offal & mixes)	wild game meat
l	5	1	1				7	1	3	1	2		7	2
h		1	2	1	1	1	6		3	1			4	4
n			1				1	2	1		3	1	7	1
p	1	1	1	1	1	1	6		1	1		1	3	
f							0	1	1	1		1	4	2
e			1	1		1	3	3	1	1			5	
o		1	2	1	1	1	6		1		1	2	4	2
c							0	1	1			1	3	
s		1	1	1			3		1		1	1	3	1
b				1	1	1	3			1		1	2	
d		1	2	1	2		6			1		2	3	
k														1
sum	6	6	11	7	6	5	41	8	12	8	7	10	45	13

* initial letter of the name of the company
